# Colorectal adenoma and carcinoma specific miRNA profiles in biopsy and their expression in plasma specimens

**DOI:** 10.1186/s13148-016-0305-3

**Published:** 2017-02-14

**Authors:** Zsófia Brigitta Nagy, Barnabás Wichmann, Alexandra Kalmár, Orsolya Galamb, Barbara Kinga Barták, Sándor Spisák, Zsolt Tulassay, Béla Molnár

**Affiliations:** 1 Molecular Gastroenterology Laboratory, 2nd Department of Internal Medicine, Semmelweis University, Szentkirályi Str. 46, Budapest, 1088 Hungary; 2 Molecular Medicine Research Group, Hungarian Academy of Sciences, Budapest, Hungary; 3 Current Address: Department of Medical Oncology, Dana-Farber Cancer Institute, Boston, MA USA

**Keywords:** microRNA, Colorectal cancer, Colorectal adenoma, miRNA profiling, Microarray, Real-time PCR

## Abstract

**Background:**

MiRNA expression markers are well characterized in colorectal cancer (CRC), but less is known about miRNA expression profiles in colorectal adenomas. Genome-wide miRNA and mRNA expression analyses were conducted through the colorectal adenoma dysplasia sequence. Furthermore, analysis of the expression levels of miRNAs in matched plasma samples was performed, focusing on biomarker candidates; miRNA and mRNA expression analyses were performed on colorectal biopsies and plasma samples (20 normals; 11 tubular and 9 tubulovillous adenomas; 20 colorectal carcinomas) by miRNA 3.0 and Human Transcriptome Array (Affymetrix) and validated by RT-qPCR. Microarray data were analyzed using Expression Console and mRNA targets were predicted using miRWALK 2.0.

**Results:**

Based on microarray analysis, 447 miRNAs were expressed in tissue and 320 in plasma. Twelve were upregulated (miR-31, 8-fold *p* < 0.001) and 11 were downregulated (miR-10b 3-fold *p* < 0.001) in neoplastic lesions compared to normal group. Eleven miRNAs showed altered expression between adenoma subtypes (miR-183 2.8-fold change, *p* < 0.007). Expression level of 24 miRNAs differed between adenoma and CRC groups (including miR-196a, 3.5-fold). Three miRNAs (miR-31, miR-4506, miR-452*) were differentially expressed in adenoma compared to normal both in tissue and plasma samples. miRNA expression data were confirmed by RT-PCR both in plasma and matched tissue samples.

**Conclusions:**

MiRNAs showed characteristic expression changes during CRC development in tissue. miRNAs were also presented in plasma and positively correlated with matched tissue expression levels. The identified miRNA expression changes could be verified RT-PCR methods facilitating routine application.

**Electronic supplementary material:**

The online version of this article (doi:10.1186/s13148-016-0305-3) contains supplementary material, which is available to authorized users.

## Background

Colorectal cancer (CRC) is the third most common malignant neoplasm worldwide [1]. Adenocarcinoma cells differ from healthy epithelial cells having morphological, epigenetic, and genetic alterations, that are reflected in the altered function of the epithelial cells in the colon. In the classic model of tumorigenesis, CRC develops through polyps to cancer, starting with an early development to adenoma from an aberrant crypt foci followed by colorectal tumor progression. This multistep process is influenced by several underlying genetic and epigenetic alterations, as Fearon and Vogelstein proposed [2].

Several studies have examined adenomas the intermediate step of colorectal carcinoma development [3, 4]. As Kinzler and Vogelstein described, tubular adenomas can transform into advanced adenomas with severe dysplasia and a high risk of malignant progression finally evolving to adenocarcinoma [5]. The adenoma-dysplasia-carcinoma sequence has been genetically well evaluated and explored by targeted and whole genome mutation analysis [6].

Analyses of multiple parallel gene expression alterations are providing deeper insights into oncogenic transformation. Several classes of screening targets have been developed in colorectal cancer: proteins [7], DNA [8], messenger RNA [9], and microRNA (miRNA) [10]. Beside mRNAs, small RNA molecules are also involved in tumor progression: the miRNAs are 19–23 nucleotide long, non-coding endogenous single-stranded RNAs that function as posttranscriptional gene regulators by binding to their target mRNAs [11]. miRNA expression alterations have been observed in various types of human cancers including breast cancer [12], hepatocellular carcinoma [13], colorectal cancer [14], and lung cancer [15].

Deregulated microRNAs have an important role in progression of colorectal cancer but less is known about their role in premalignant adenomas. Changes in the expression pattern of miRNAs can be informative and highly significant in the colorectal adenoma-carcinoma sequence progression as well. However, to date precancerous lesions (such as adenomas) have been investigated less frequently than cancers compared to healthy tissues [16, 17].

Possible miRNA-mRNA interactions can be predicted using in silico prediction methods, that became [18] available using new (computer) algorithms and techniques [19–21] which have made a more detailed analysis and prediction possible [22] and improved knowledge about cancer development.

During colorectal cancer development, release of nucleic acids (DNA, mRNA, miRNA) can be observed in blood in free circulating, protein, and exosome bound forms. Circulating miRNAs can be detected in plasma, as well [23]. Due to the stability of miRNAs, they can be used as stable prescreening molecules in plasma samples, once their sensitivity and specificity is proven. Microarray technology allows characterization of tissue samples by screening the expression level of thousands of genes simultaneously [24]. Recently, several studies have been published using microarray platforms in addition, real-time PCR array methods are already available to enable systematic, whole genome miRNA expression analysis in comparison between normal and tumor samples [4, 25, 26].

To date our research group has screened systematic alterations of DNA methylation [27], mRNA expression [28–30], and protein expression [31] in colorectal cancer development. Furthermore, the Septin 9 plasma methylation marker was also evaluated [32]. Analysis of miRNAs, as part of an epigenetic regulatory network complements our developing understanding of this complex process.

The aim of the present study was to perform an additional global miRNA microarray analysis of tubular and tubulovillous adenoma biopsy specimen completed with colorectal adenocarcinomas using fresh frozen and FFPE tissue. Changes in miRNA expression patterns were also investigated from plasma samples obtained from the same patients. Furthermore mRNA analysis was also performed using Human Transcriptome Arrays 2.0, to allow integrated investigation of the genome-wide parallel mRNA and miRNA expression changes.

## Methods

### Clinical samples

The study protocol was approved by the local ethics committee (Semmelweis University Regional and Institutional Committee of Science and Research Ethics; Nr.: ETT TUKEB 23970/2011), and written informed consent was provided by all patients. Patients did not receive chemo- or radiotherapy prior to sample collection. Clinicopathological features can be found in Additional file 1: Table S1.

Fresh frozen colonic biopsy samples (approx. 3–5 mg tissue specimens) from 20 colorectal cancer 20 normal colorectal, 11 tubular and 9 tubulovillous adenoma tissues were obtained during routine colonoscopy examinations. Tissues were immediately placed in RNALater (Qiagen, cat. no. 76104) and were stored at −80°C. FFPE specimens were also collected and processed from parallel surgical tissues. EDTA-stabilized peripheral blood samples were obtained after approved consent (Table 1).

**Table 1:** Samples used in the study

Sample types	Patients groups				Methods
	Normal	Tubular adenoma	Tubulovillous adenoma	Adeno-carcinoma	
FF tissue biopsy	*n* = 20	*n* = 11	*n* = 9	*n* = 20	Microarray
Matched plasma	*n* = 4	*n* = 4	*n* = 4	*n* = 4	Microarray
FF tissue biopsy (pooled)	Pooled tissue samples				Real-time PCR
	*n*1 = 1–10 *n*2 = 11–20	*n*1 = 1–11	*n*1 = 1–9	*n*1 = 1–10 *n*2 = 11–20	
Matched plasma	*n* = 3	*n* = 3	*n* = 3	*n* = 3	Real-time PCR
FFPET surgical tissue	*n* = 3	*n* = 3		*n* = 3	Real-time PCR

*FF* fresh frozen, *FFPET* formalin-fixed, paraffin-embedded tissue

### Isolation of total RNA including miRNA from fresh frozen biopsies

Total RNA including miRNA was isolated from biopsy samples using the High Pure miRNA Isolation Kit (Life Science Roche, cat. no. 05080576001) according to the manufacturer’s instruction, after homogenization with MagNA Lyser instrument (Life Science Roche, cat. no. 03358976001). RNA integrity was evaluated using 2100 Bioanalyzer (Agilent, cat. no. G2940CA), and RNA yield was quantified using Qubit 1.0 fluorometer (ThermoFisher Scientific).

### Total RNA isolation from FFPE specimen

Hematoxylin and eosin stained sections (10 μm) from each formalin-fixed paraffin-embedded tissue blocks were used for histological analysis.

Three tissue sections were cut from standard formalin-fixed paraffin-embedded blocks and were transferred to a microcentrifuge tube per isolation. Deparaffinization was performed with 1 ml xylene for 10 min twice and washing with 1 ml absolute ethanol for 10 min twice. miRNAs were isolated from three air-dried deparaffinized sections per isolation using the High Pure miRNA Isolation Kit.

### miRNA isolation from plasma specimen

Peripheral blood samples were drawn into EDTA-containing tubes and centrifuged at 1350 rpm for 12 min at 24 °C (room temperature). The supernatants were then centrifuged with the same parameters in a second round. The plasma samples were stored at −20 °C until use. The miRNA fraction was extracted using the QIAamp Circulating Nucleic Acid Kit (Qiagen, cat. no 55114) with the following protocol: 1 ml of plasma was mixed with 100 μl proteinase K (Qiagen, cat. no. 55114) and 800 μl ACL buffer (Qiagen, cat. no. 55114), and samples were incubated at 60 °C for 30 min. Then 1800 μl ACB Buffer (Qiagen, cat. no. 55114) and 3 ml isopropanol were added to the mix, and the tubes was gently inverted a few times. Tubes were then incubated at −20°C overnight. The mixture was filtered through the column followed by washing steps with 1 ml 70% and 1 ml absolute ethanol. RNA was eluted in 30 μl puffer AVE (Qiagen, cat. no.55114).

### Tissue and plasma miRNA microarray and mRNA expression profiling by Human Transcriptome Array 2.0

The miRNA expression profiles were analyzed by GeneChip miRNA 3.0 array (Affymetrix, cat. no.902413). 1 μg of total RNA including miRNA from tissue, and the total amount of eluted miRNA fraction from plasma samples were biotin-labeled using the Flashtag Biotin HSR RNA Labeling Kit (Affymetrix, cat. no.901911). The samples were hybridized for 16 h in a GeneChip Hybridization Oven 645 (Affymetrix, cat. no. 00-0331), then were washed and stained using a GeneChip Fluidics Station 450 (Affymetrix, cat. no. 00-0079) with the FS450_0002 fluidics protocol and scanned with a GeneChip® Scanner 3000 7G (Affymetrix, cat. no.00-0210).

Out of the 60 fresh frozen biopsy tissue samples, 20 (7 normal, 2 tubular, 4 tubulovillous adenoma, and 7 tumor) were selected for mRNA expression analysis using a GeneChip Human Transcriptome Array 2.0 (Affymetrix, cat. no.902162) according to the manufacturer’s instructions.

### Real-time quantitative PCR array analysis from fresh frozen and individual FFPE tissue specimen

40 ng of total RNA including miRNA was reverse transcribed using the miRCURY LNA™ Universal RT cDNA Synthesis Kit (Exiqon cat. no. 203301). The cDNA template was then amplified using the microRNA Ready-to-Use PCR, Human Panel I + II (Exiqon) in 384-well plates according to the manufacturer’s instruction. The qPCR reactions were run on a LightCycler 480 System (Life Science Roche) using the thermal-cycling parameters recommended by Exiqon (Denaturation at 95 °C 10 min, 45 amplification cycles at 95 °C 10 s 60 °C 1 min). The amplification curves were analyzed by LightCycler 480 Software (Life Science Roche, cat. no. 04994884001). From the 768 wells, 742 miRNA primer sets were used for miRNA expression profiling, there remaining wells contained interplate calibrator oligonucleotides, spike-in control oligonucleotides, and empty wells. Hsa-miR-423-5p was used for normalization. The relative quantification of miRNA expression levels was performed using delta deltaCp method [33].

### In silico miRNA-mRNA target prediction

Three miRNA were selected: miR-31 has the highest fold change in normal vs. adenoma normal vs. CRC comparison; miR-4417 and miR-497 were one of the continuously upregulated or downregulated miRNAs in adenoma-carcinoma transition and their mRNA targets were predicted using five algorithm: TargetScan, miRanda, PICTAR2, RNAHybrid and miRWalk on miRWalk 2.0 platform. Using DAVID tools (The Database for Annotation, Visualization and Integrated Discovery v6.7) we acquired pathway enrichment from gene ontology. Through the KEGG pathway databases, we examined the pathway target enrichment (*p* < 0.01) of selected groups of miRNAs according to Yin et al. [34].

### Immunohistochemistry

Immunohistochemistry for Cyclin D1 was performed on formalin-fixed and paraffin-embedded tissue samples from normal (*n* = 15) adenoma (*n* = 15) and CRC (*n* = 10) patients (all samples diagnosed in H&E serial sections by an expert pathologist). Following deparaffinization and rehydration, microwave-based antigen retrieval was performed in TRIS EDTA buffer (pH 9.0) (900 W/10 min, then 340 W/40 min). Samples were immunostained with Cyclin D1 monoclonal antibody (clone SP4: Histopathology Ltd., Hungary, cat no. 10074, 1:100 dilution) and diaminobenzidine-hydrogen peroxidase–chromogen substrate system (HISTOLS-DAB, Histopathology Ltd., cat. no. 30014.K) were used. Slides were digitalized by Pannoramic 250 Flash II scanner (3DHISTECH Ltd.), and digital slides were semi-quantitatively analyzed with Pannoramic Viewer (ver.:1.15.3; 3DHISTECH) based on *Q* score method (scored by multiplying the percentage of positive cells (P) by the intensity (*I*: +3, +2, +1, 0). Formula: *Q* = *P* × I; maximum = 300). Primarily we examined separately the epithelial and stromal compartments, then summarize (Σ) these scores (ΣQ score maximum: 600) in order to remain comparable with our miRNA and mRNA expression analyses using whole biopsy samples.

### Statistical analysis

Probe cell intensity files of microarrays were analyzed using Expression Console Software (Affymetrix). The robust multichip averaging (RMA) algorithm was used for background correction normalization and probe level summarization. GeneChip miRNA 3.0 array contains probe sets for 1733 mature miRNAs. Human Transcriptome Array 2.0 files were analyzed using Expression Console Software and Transcriptome Analysis Console (Affymetrix). Values of *p* < 0.05 were considered as statistically significant with a logFC > |1|.

## Results

### Identification of expressed miRNAs in diagnostic groups

The absolute numbers of expressed miRNAs in the analyzed sample groups were determined based on the intensity values of oligonucleotide probes for 1733 human mature miRNAs that are synthesized on the surface of GeneChip miRNA 3.0 arrays. Present values (based on hybridization) were calculated by Expression Console Software (Affymetrix) using the statistical present/absent calls method.

**Fig. 1:**
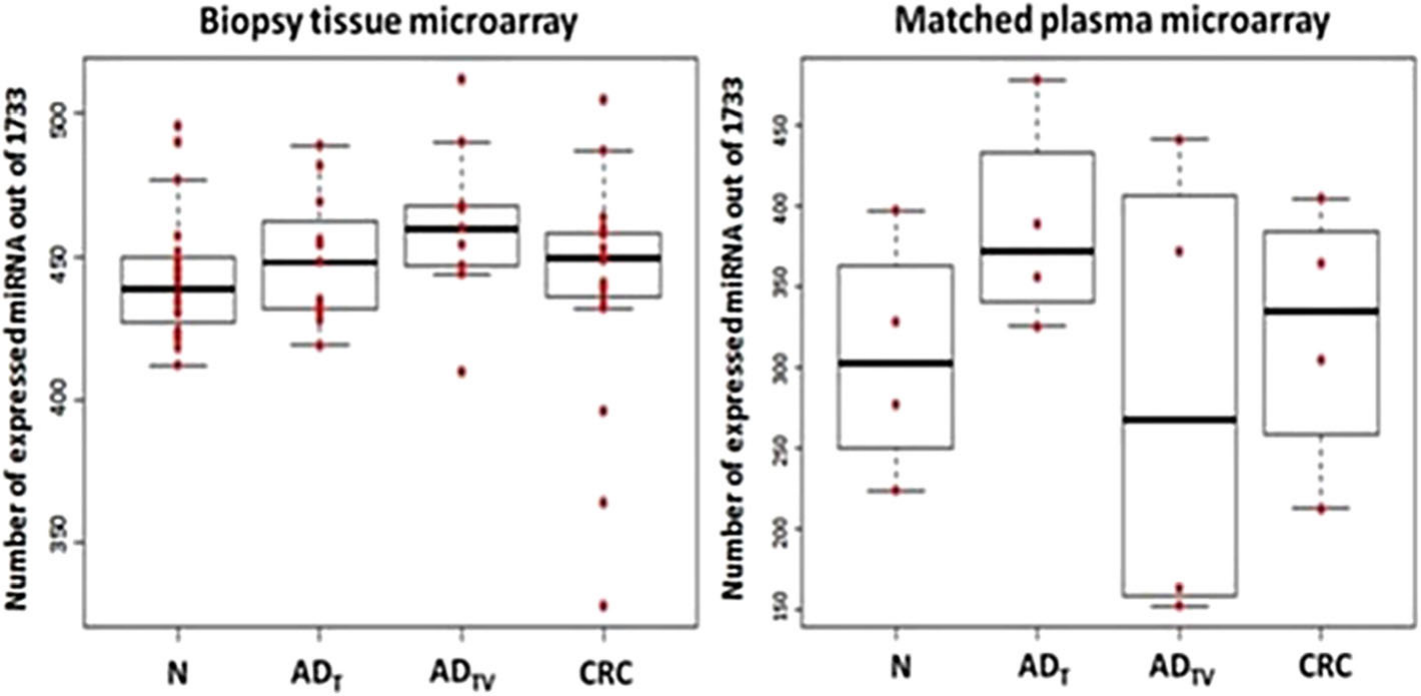
Number of miRNAs (considered as present) expressed in tissue and plasma samples. *N* normal*; AD*_*tub*_ tubular adenoma; *AD*_*tubvill*_ tubulovillous adenoma; *CRC* colorectal cancer

From the 1733 mature miRNA probe sets, 442 had positive values in normal tissue samples, 460 present values were detected in adenoma (tubular and tubulovillous) tissue samples, and 441 present values were detected in colorectal carcinoma tissue samples (Fig. 1). There were no significant differences in detection calls between the diagnostic groups. The present/absent calls in matched plasma samples from the same diagnostic groups were also observed, 306 miRNAs were observed in the plasma of normal subjects, 334 miRNAs in the plasma of adenoma patients, and 321 miRNAs in the plasma of patients with colorectal cancer (Fig. 1).

### miRNA expression in different patient groups

#### Microarray analysis

**Fig. 2:**
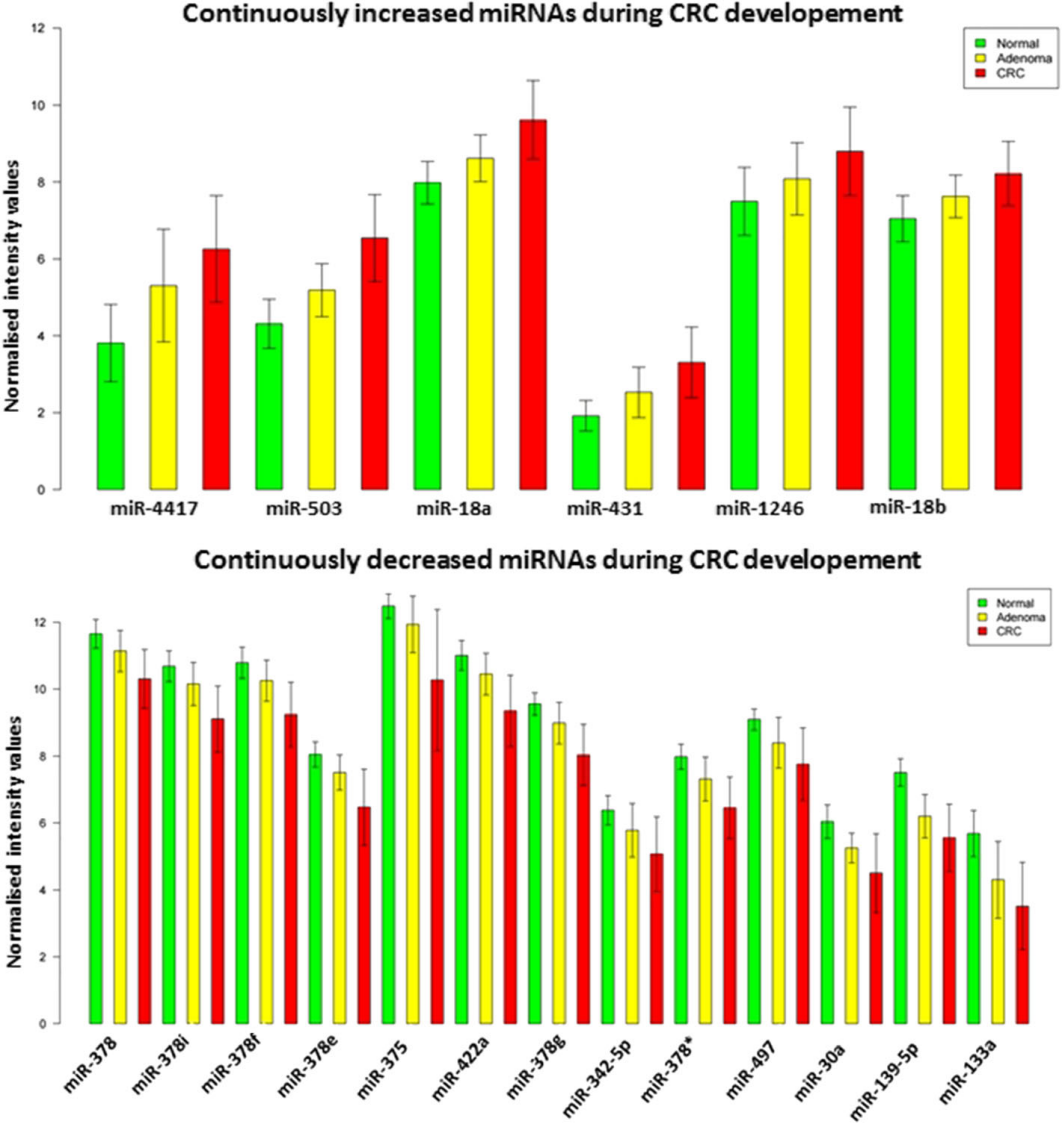
*Bar charts* showing the continually upregulated and downregulated miRNAs during cancer progression based on microarray data in tissue samples. *Green* color represents healthy tissues, *yellows* are adenomas, *red* colors are colorectal cancer samples (*p* < 0.05, logFC > |0.5)|

miRNA microarray analysis was performed on biopsy tissue samples from healthy patients and also from patients with tubular or tubulovillous adenoma or colorectal cancer. Along the colorectal adenoma-carcinoma transition, based on our microarray profiling 19 continuously upregulated or downregulated miRNAs were selected (Fig. 2). Expression elevation could be observed in case of miR-4417 which was upregulated 2.8-fold in adenoma compared to normal samples, moreover, this expression level was further elevated (1.9-fold) in CRC samples. Interestingly, miRNA-378 family (-i;-f;-e;-g;-*) members are represented highly in downregulated groups of miRNAs. Eight (without miR-378 variants) downregulated miRNAs showed approx. 1.4-2.4-fold lower expression in adenomas compared to healthy, and reduction was 1.5-3-fold lower in carcinoma samples (Fig. 2).

**Fig. 3:**
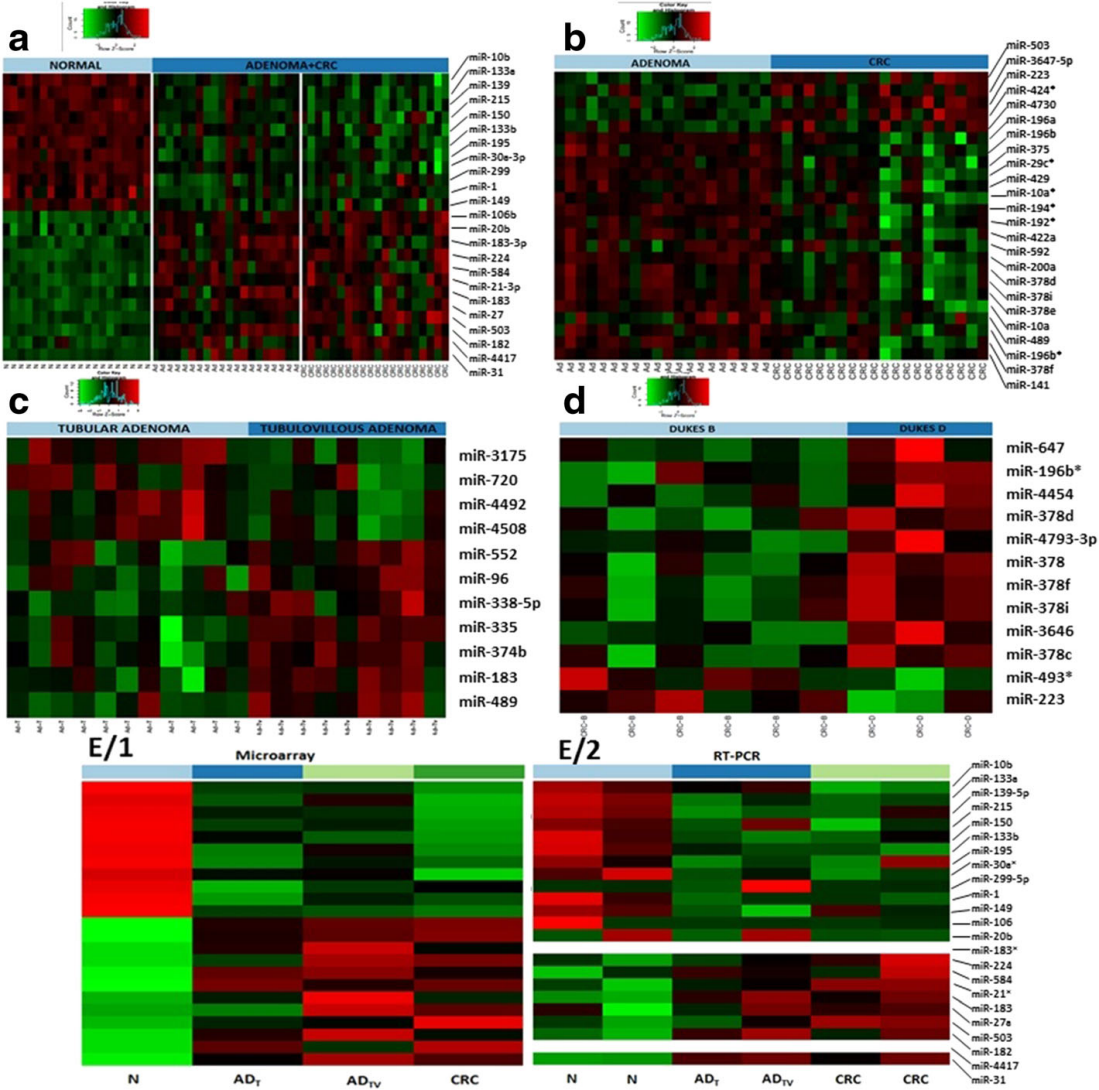
**a** Heatmap showing differential miRNA expression in diseased vs normal control group. *Green* color indicates lower than mean intensity, and *red* represents higher than mean intensity. Each *row* represents a miRNA and each column represents a sample (*p* < 0.05, logFC > |1)|. **b** Comparison of tubular and tubulovillous adenoma with colorectal cancer tissue samples. Each *row* represents a miRNA. Each *column* represents a tissue sample. Selected miRNAs are sorted by statistical conditions: *p*< 0.05, logFC > |1|. **c** Comparison of tubular and tubulovillous adenoma samples. Each *row* represents a miRNA. Each *column* represents a tissue sample. Selected miRNAs are sorted by statistical conditions: *p*< 0.05, logFC > |1|. **d** Cluster analysis of Dukes B and D stages. Results based on three miRNAs showed significant expression alteration between groups (*p* < 0.05). **e** Real-time PCR (E/2) validation of microarray (E/1) results in fresh frozen biopsy tissue samples. *N* normal; *AD* tubular adenoma; *AD*_*TV*_ tubulovillous adenoma; *CRC* colorectal cancer

Figure 3a represents a heatmap of microarray data describing the miRNA expression profiles in precancerous and neoplastic lesions vs. normal samples. Twenty-three miRNAs (11 downregulated and 12 upregulated) were significantly differentially expressed in healthy normal colonic tissue vs. precancerous and neoplastic lesions. The expression of all 12 upregulated miRNAs were increased at least 2-fold in tissues from the normal-adenoma-carcinoma sequence progression. The highest miRNA expression alteration was observed in case of miR-31 showing eightfold higher expression both in adenoma and in CRC tissue compared to normal samples (Additional file 2: Table S2).

24 miRNAs showed characteristic differences between adenoma and colorectal cancer samples. Only five miRNAs were upregulated in colorectal cancer compared to adenoma (Fig. 3b).

When we selected miRNAs to characterize tubular vs. tubulovillous adenoma, 11 miRNAs showed altered expression between the adenoma subgroups. miR-489 expression showed the greatest difference between adenoma subtypes with a > 4.5-fold increase in tubulovillous adenoma samples. The fold change of other miRNAs with significantly altered expression were in the range of 2–4 (Fig. 3c).

By the analysis of microarray data, 12 differentially expressed miRNAs could be detected between different stages of colorectal cancer (Fig. 3d). The majority of miRNAs showed overexpression in Dukes D stage samples. Interestingly, members of miR-378 family were highly represented in this comparison, as well as in the groups with continuously changing expression in adenoma to carcinoma progression.

**Fig. 4:**
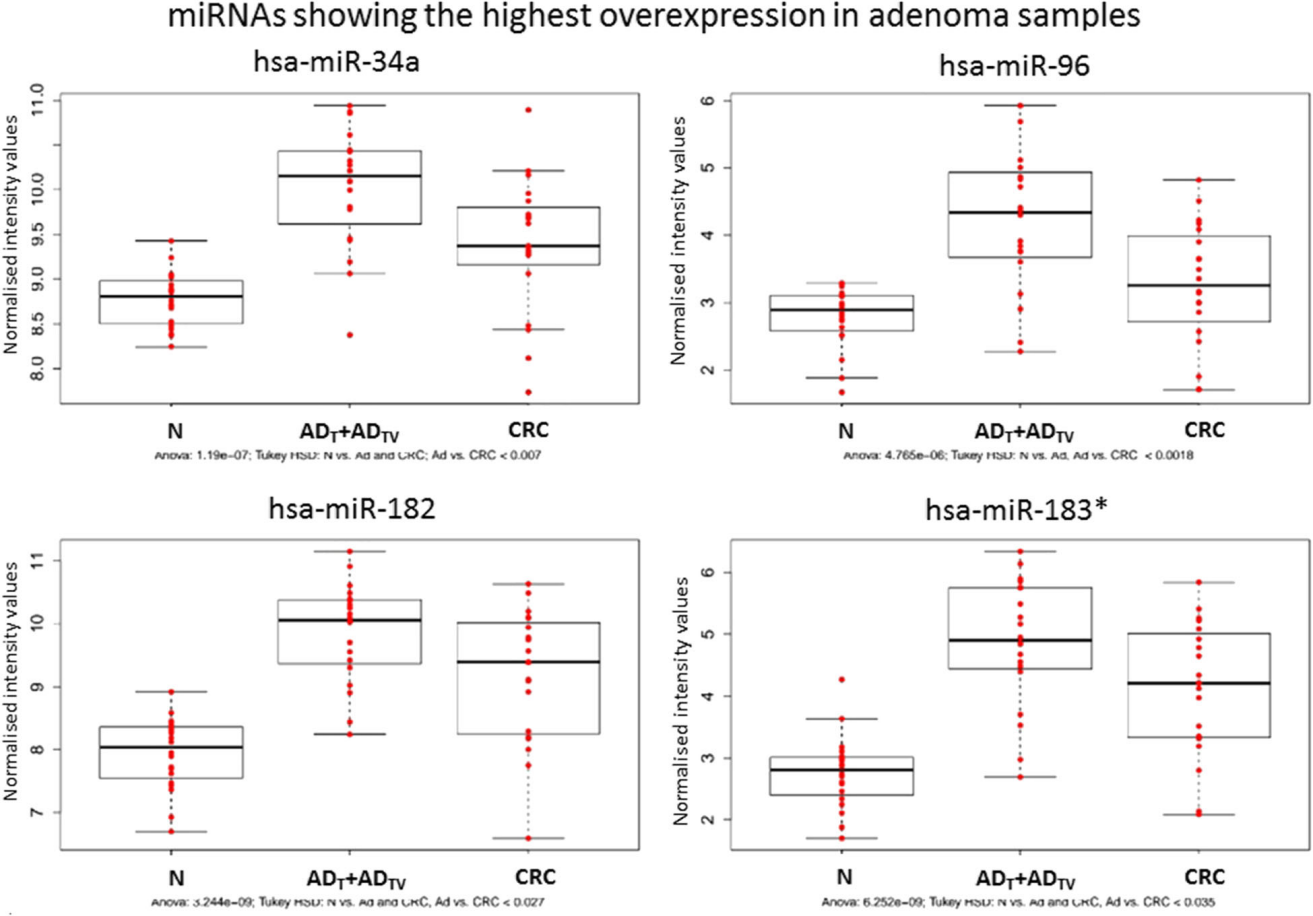
miRNAs showing the highest overexpression in adenomas (but then downregulated in carcinoma). *N* normal; *AD* tubular adenoma; *AD*_*TV*_ tubulovillous adenoma; *CRC* colorectal cancer

Interestingly, most of the differentially expressed miRNAs in the adenoma-CRC comparison were upregulated in adenomas compared to CRC samples (Fig. 3b). Therefore, we focused on these adenoma-specific miRNA groups and selected those ones which are upregulated in adenomas but after significantly downregulated in CRC patients. Figure 4 represents four miRNAs showing the highest overexpression in adenoma samples. Levels of miR-182, miR-183*, miR-96, and miR-34a are decreased 1.5-fold or higher in carcinoma tissue samples compared to adenoma.

### miRNA microarray validation

In order to confirm the accuracy and reliability of the microarray data, the same tissue/RNA samples as used in miRNA microarray analysis were analyzed using the miRCURY Human Panel real-time PCR (Exiqon) containing 742 mature miRNA oligonucleotides. RT-PCR validation was done on four tissue samples pooled (with equal ng of RNA of the samples in each group) according to the analyzed diagnostic groups (normal, CRC, tubular adenoma, and tubulovillous adenoma).

miRNAs showing altered expression in precancerous and cancerous lesions by microarrays were selected, and the expression tendencies were detected by real-time PCR validation (Fig. 3e). The results of the analysis confirmed the majority of our microarray data, except for six miRNAs: miRNA-4417 was not represented in the PCR panels, miRNA-183* assay did not give any signal, and four miRNA assays including miR-299-5p, miR-20b, miR-195, and miR-215 had outlier signals in pooled PCR patient groups.

### miRNA target prediction and validation on Human Transcriptome Arrays

**Fig. 5:**
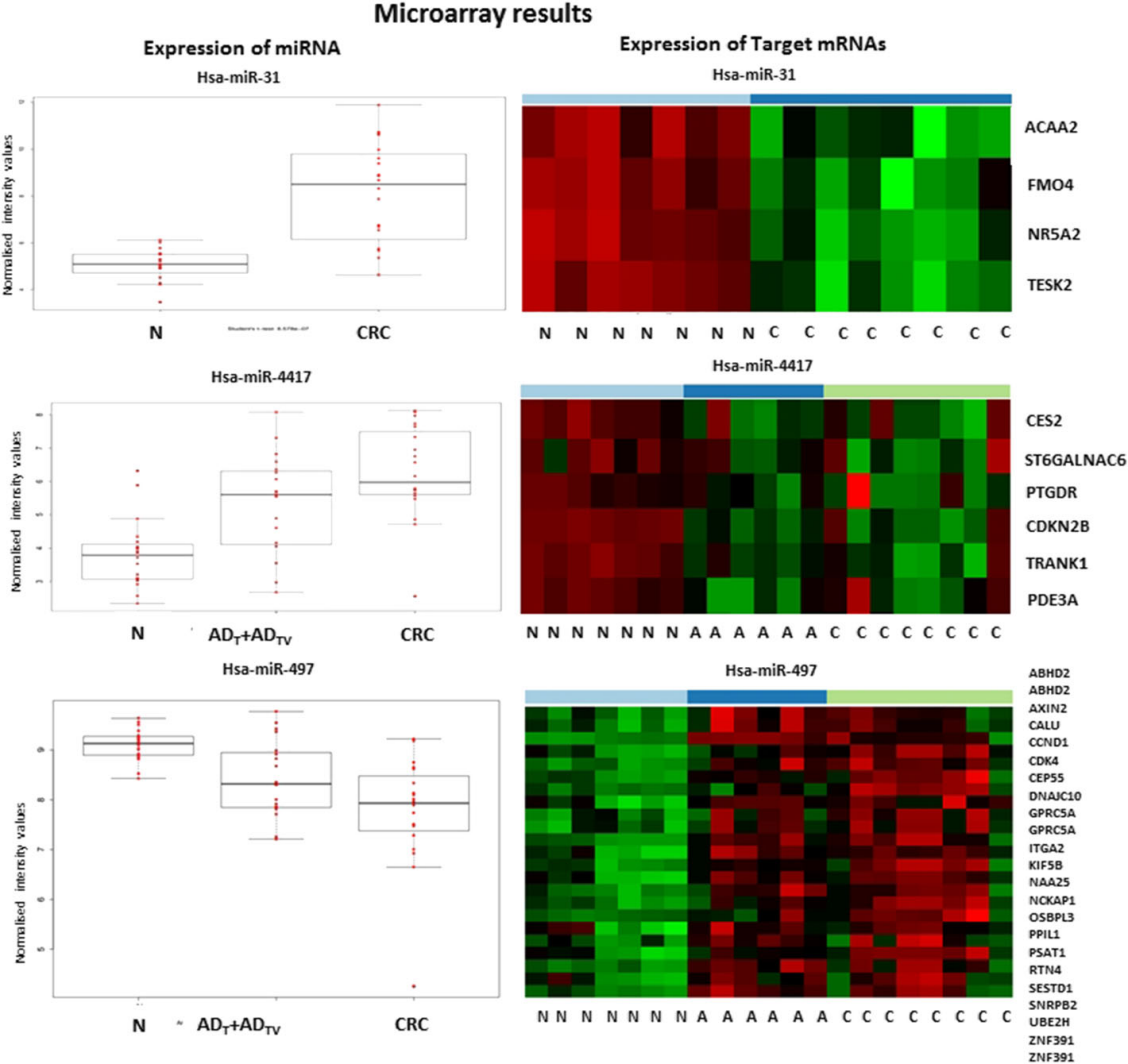
Selected miRNAs with continuous expression alteration along the colorectal adenoma-carcinoma sequence. Left box plots represent the miRNA expression in tissues. Heatmaps represent the target mRNA expression in tissues. *N* normal; *A* adenoma; *CRC* colorectal cancer. (see full names of genes are in list of Abbrevations)

Based on the Human Transcriptome Array 2.0 mRNA expression results, downregulated mRNA targets were selected. Three miRNAs were selected: miR-31 has the highest fold change in normal vs. adenoma, normal vs. CRC comparison; miR-4417 and miR-497 were one of the continually upregulated or downregulated miRNAs in adenoma-carcinoma transition. mRNA targets were predicted on miRWalk 2.0 platform in silico. Parallel analysis on Human Transcriptome Arrays representing > 245,000 coding transcripts was performed. The selected mRNA targets of the miRNAs can be seen on Fig. 5. The mRNA expression pattern was observed inverse compared to the miRNA expression pattern.

### Deregulated miRNAs’ biological function and pathway analysis by KEGG and GO database

Target genes of the continuously overexpressed and underexpressed miRNAs during CRC development (without miR-378 homologues) (Fig. 2) participate in pathways related to signal transduction (PI3K-Akt KEGG pathway ID: hsa04151, MAPK-KEGG pathway ID: hsa04010) and cancer (KEGG pathway ID:hsa05200) from the top 24 selected KEGG pathways. GO analysis was also conducted on these miRNAs revealing transcription regulations and cell proliferations. (Additional file 3: Tables S3 and S4). Target genes of four miRNAs showing the highest expression in adenomas (Fig. 4) could be related to the following pathways: microRNAs in cancer (ID: hsa05206), pathways in cancer (ID: hsa05200) (Additional file 3: Tables S7 and S8). These predicted genes are involved mostly in DNA transcription regulation and cell proliferations.

### Analysis of fresh frozen tissue samples and FFPE surgical samples by real-time PCR

**Fig. 6:**
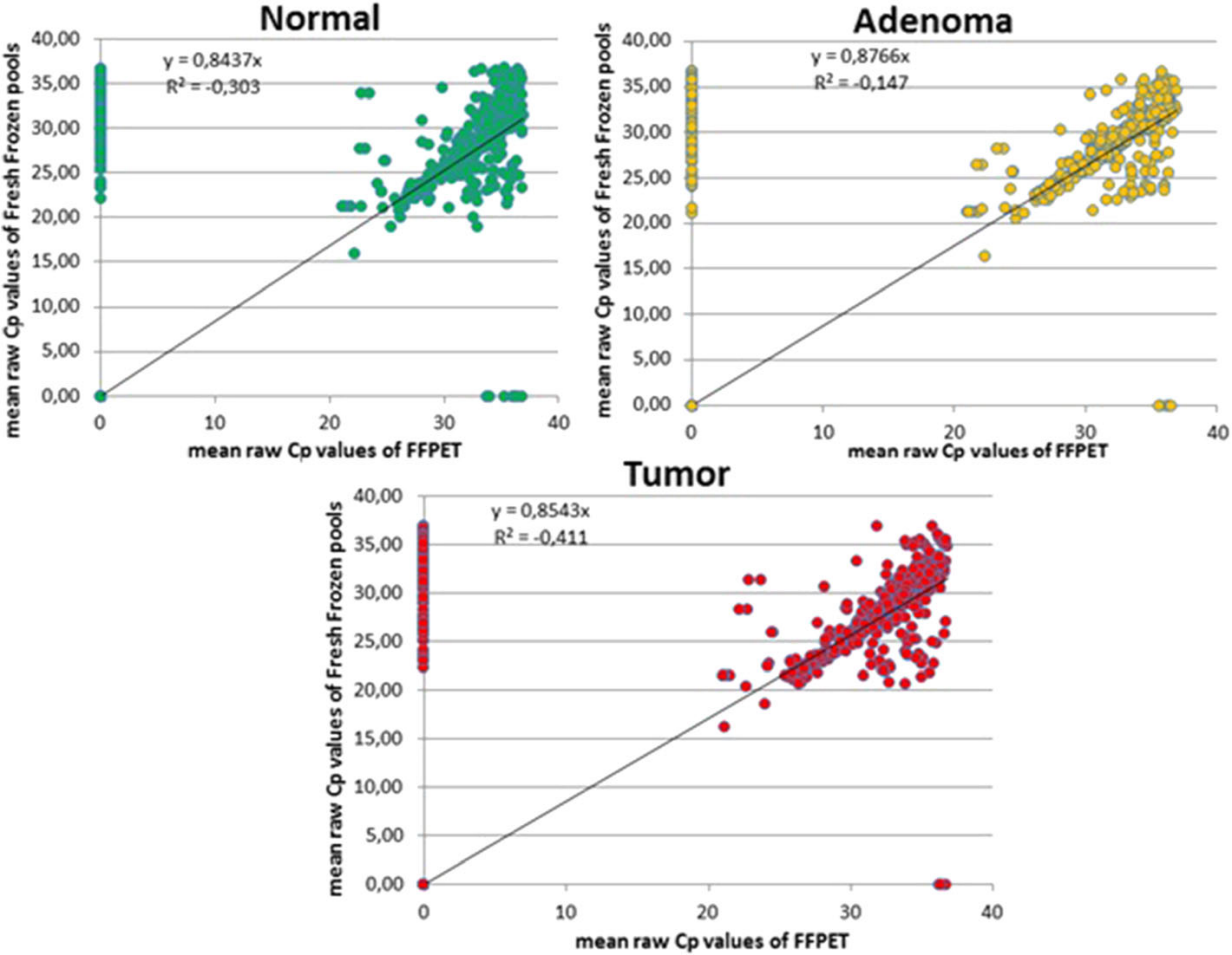
Correlation between miRNA expression in formalin-fixed, paraffin-embedded (FFPE), and fresh frozen (FF) colonic tissue. dCP values were illustrated in graphs. Axis of *X* contains all the detected raw Cp values of each miRNA in case of FFPE tissues, axis of *Y* contains all the detected raw Cp values in case of fresh frozen tissue samples

Delta Cp values of pooled fresh frozen tissue samples and FFPE tissue samples were visualized on graphs (Fig. 6). The expression of most miRNAs slightly correlated in fresh frozen and FFPE tissue types. These miRNAs could be abundant and are less affected by tissue processing (miR-135b, miR-196b, miR-31). There were some miRNAs whose expressions were more influenced by the sample processing types (FF or FFPE). Expectedly, miRNAs can be isolated more efficiently from fresh frozen samples than from paraffin embedded tissues, however, about more than 60% (N-67%, A-65%, CRC-71%) of miRNAs expressed in FFPE tissues. Interestingly, among the 742 miRNAs represented on the Exiqon Human Panel I + II PCR plates, 50% (N-44%, A-44%, CRC-48%) could be detected only in fresh frozen samples but not in FFPE tissues.

### Microarray results of matched plasma samples

**Fig. 7:**
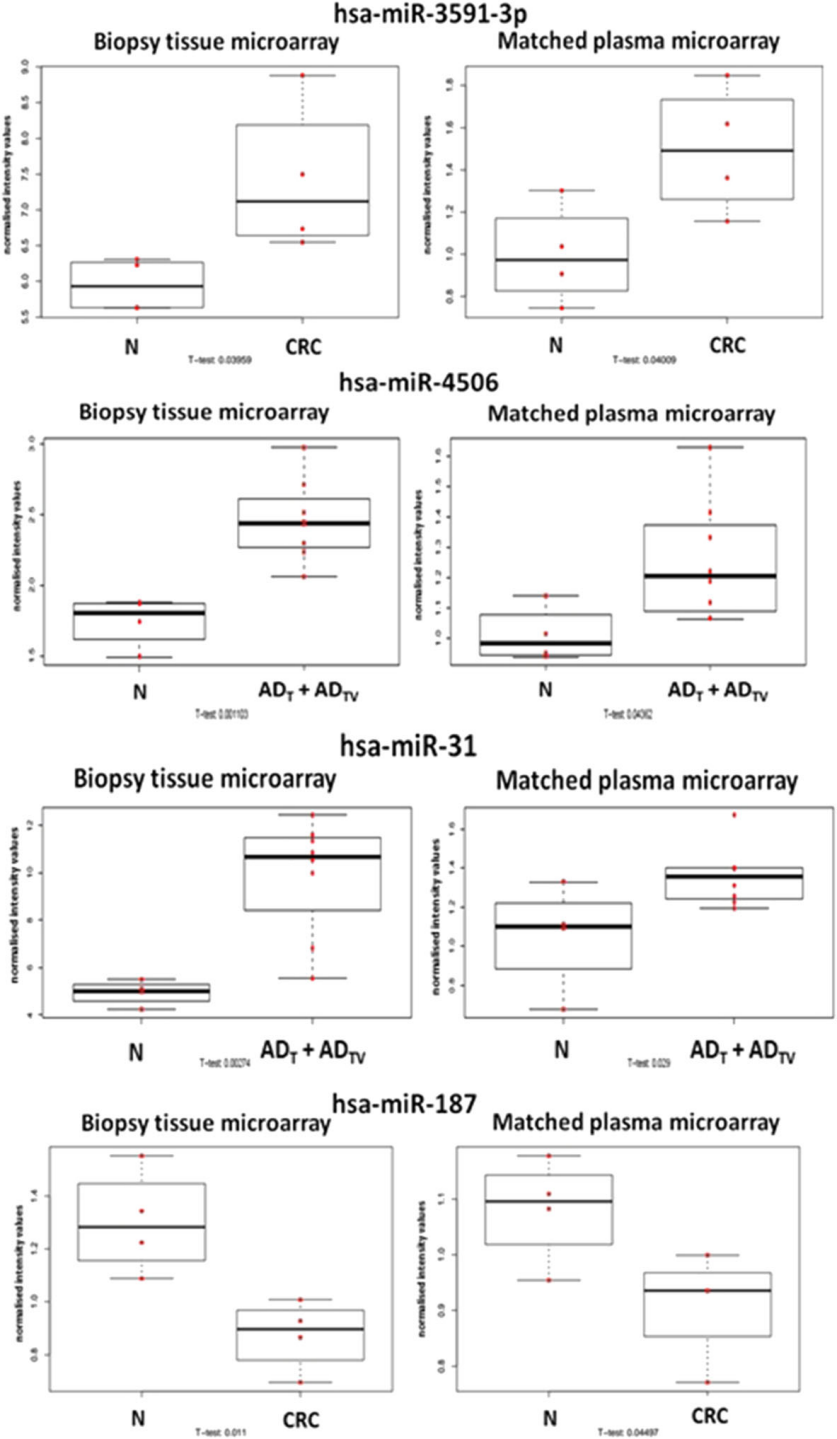
miRNA expression shows the same tendency in tissue and plasma samples. *N* normal; *AD* tubular adenoma; *AD*_*TV*_ tubulovillous adenoma; *CRC* colorectal cancer

To investigate the clinical potential of miRNAs detected in tissue, plasma samples were collected from the same patients and miRNA microarray analysis was performed. Four miRNAs were found to be expressed in both tissue and plasma samples moreover, in each sample type the selected miRNAs showed the same expression tendency with miRNAs showing altered expression between diagnostic groups (visualized by boxplots) (Fig. 7).

#### Immunohistochemistry of Cyclin D1

**Fig. 8:**
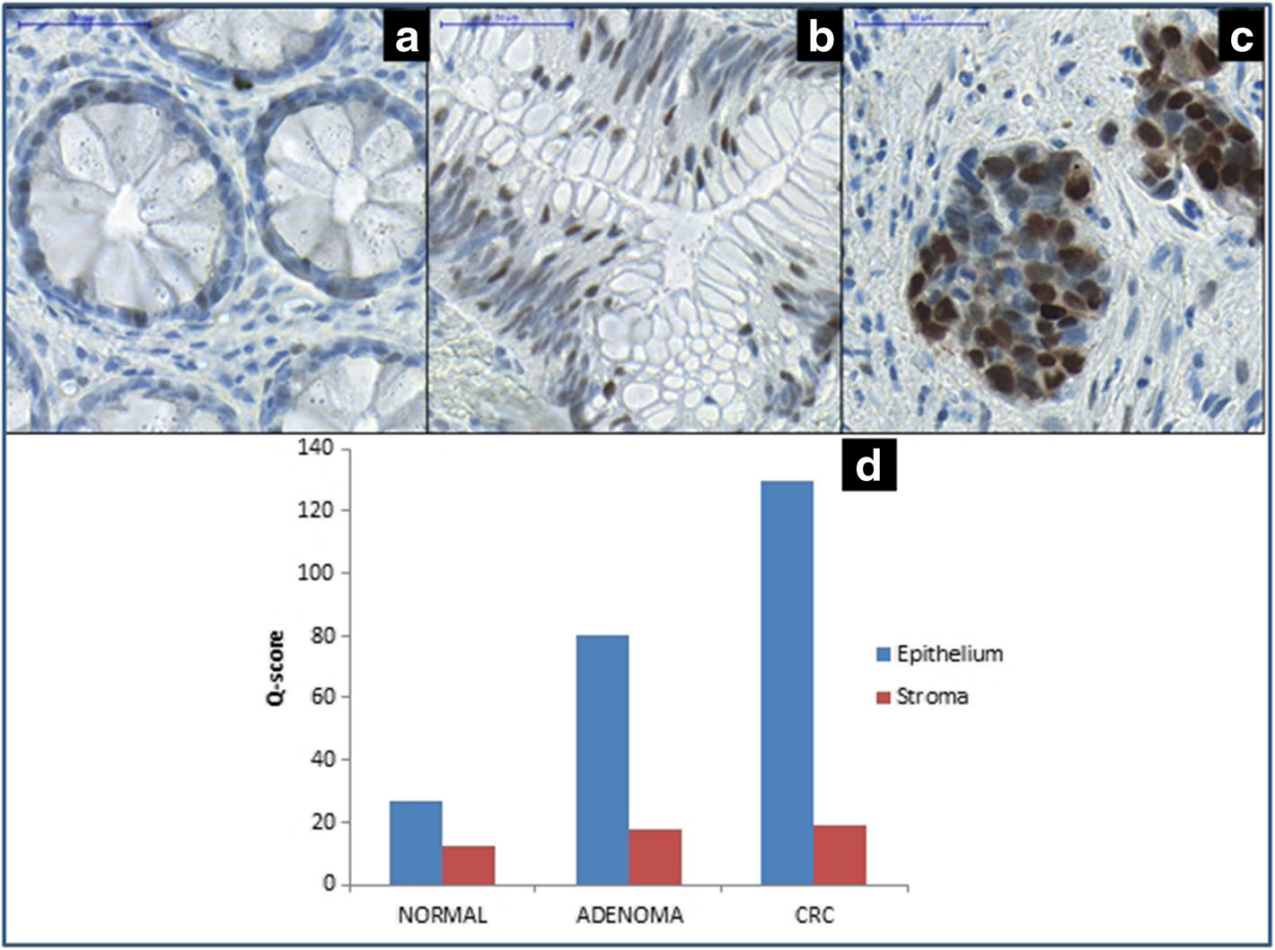
The expression of Cyclin D1 in normal epithelium (**a**), adenoma (**b**), and CRC (**c**) samples. Digital microscopic image; 50x magnification; *scale bar*: 50 μm. The detailed *Q* scores of examined sample groups are illustrated (**d)**

The normal epithelium showed low nuclear Cyclin D1 expression (representative scoring values: 0; +1; *Q* score: 26.66 ± 8.16), whereas among all stromal cells only few showed strong immunoreaction, (+2 and +3) (*Q* score: 12.67 ± 3.19 and ΣQ score: 39.32 ± 8.63; Fig. 8a). The stromal protein expression was low, but significantly (*p* < 0.05) increased in adenomas (*Q* score: 17.50 ± 5.77) and in CRCs (*Q* score: 19.00 ± 7.34). Heterogenic, significantly (*p* < 0.05) increased nuclear Cyclin D1 expression (representative scoring values: +2; +3) was detected in epithelial compartment of adenomas (*Q* score: 80.00 ± 17.88; Fig. 8b) and CRCs (*Q* score: 129.50 ± 45.85; Fig. 8c). The detailed *Q* scores of examined sample groups are illustrated on (Fig. 8d).

The detected total protein level alteration along the adenoma-carcinoma transition of the analyzed colorectal tissue samples was fundamentally constituted by the increased epithelial cyclin D1 expression (adenoma ΣQ score: 96.25 ± 18.75 and CRC ΣQ score: 147.00 ± 48.94. (N vs. Ad and Ad vs. CRC: *p* < 0.05)).

## Discussion

In this study, we have carried out a high throughput screening of microRNA expression alterations by microarray analysis in tubular, tubulovillous colorectal adenoma, and adenocarcinoma samples. Healthy colonic tissue samples and parallel plasma samples were also evaluated from the same patients. The numbers of expressed miRNAs between the different patients groups are nearly balanced, although less miRNAs were expressed in plasma samples compared with matched tissue samples (approximately 300–330 miRNAs/samples group). Slattery et al. also found less than 600 expressed miRNAs in colonic tissues on a different microarray platform (Agilent) [3]. Leidinger et al. found that average number of detected miRNAs in control plasma samples was 331, and the overall number of detected miRNAs in plasma of lung cancer patients was between 264 and 364 from a set of 1205 miRNAs analyzed [35].

Genome-wide miRNA expression-profiling studies using high throughput technologies frequently apply comparisons of normal-adenoma or normal-carcinoma pairs (Additional file 1: Table S1). Here, we have performed a comparison of control normal vs. precancerous and neoplastic lesions (adenomas and tumor samples). We have focused on those short RNAs which are continuously upregulated or downregulated through adenoma-carcinoma progression. The continuous downregulation observed in adenoma-carcinoma samples in case of miR-375, miR-378, miR-139-5p, miR-133a, and miR-422a was confirmed by others [36–44]. MiR-378 could inhibit tumor growth and invasion partly by targeting vimentin in colorectal cancer [39]. Functional analysis of miR-133a revealed that it can inhibit CRC cell growth and metastasis by targeting LIM and SH3 protein. It represses the MAPK pathway as well, as shown after predicted KEGG pathway analysis [43]. Lower expression of miR-422a was associated with advanced stages of colorectal cancer thus proving its tumor suppressive role [44].

MiR-503 and five other miRNAs (miR-4417, miR-18a, miR-431, miR-1246, and miR-18b) were the only short RNAs, which showed significant upregulation through the normal to  adenoma (dysplasia) transition in our analysis. Selected miRNAs have a role in tumor progression, as e.g., miR-503 directly targets L1 adhesion molecule (L1CAM) [45] and E2F transcription factor 3 (E2F3) [46] mRNAs involved in tumor progression. Out of the four adenoma-specific miRNAs (miR-34a, miR-96, miR-182, miR-183*) miR-96, miR-182 expression changes were proven in adenoma and carcinoma samples by Wang et al. in a deep sequencing study with a real-time PCR validation [47]. Furthermore, miR-182 has been reported to suppress ENTPD5 expression  which is involved in energy metabolism. Previously, a decreased expression of  ENTPD5 mRNA and protein levels was observed during adenoma carcinoma transition [48, 49].

In our study, strong emphasis was placed on exploration of altered miRNA expression patterns in the early benign lesions including the comparison of different adenoma subtypes. Among others, we observed > twofold decreased expression of miR-3175, miR-720, miR-4508 and miR-4492 in tubulovillous adenoma compared  to tubular adenoma samples. Of interest, miR-3175 expression was also reported to be reduced in gastric adenocarcinoma cell line [50]. Interestingly, elevated miR-720 levels were found in both adenoma subtypes compared to healthy tissue samples. However, expression levels also differed significantly between adenoma subgroups. Nevertheless, an early increasing expression of miR-720 was observed through normal to adenoma transformation, its concentration was more than 2-fold lower in tubulovillous compared to tubular adenoma tissues. Schopman et al*.* reported that miR-720 is not a classic miRNA, but rather a fragment of a tRNA [51]. It is also known as a novel serum biomarker of CRC [52]. Interestingly, miR-489 was found to be the most overexpressed miRNA between adenoma subtypes though its downregulation was observed in CRC and was also associated with other tumor types (Additional file 1: Table S1) [53, 54]. From the 24 miRNAs which could differentiate between adenoma-carcinoma, the overexpressed miR-223 was a validated *oncomiR* in CRC invasion and metastasis [55].

In this study, we also demonstrated that miRNA detection efficiency differed in distinct sample types such as fresh frozen and FFPE samples. Interestingly, more miRNA signals could be observed in FFPE than in fresh frozen tissues. Differences in the optimization of isolation protocols or application of pooled samples in case of fresh frozen tissues could cause this phenomenon.

We also analyzed the appearance of tissue miRNA expression changes in peripheral blood. In case of several miRNAs, a positive correlation was found between their expression in tissue and plasma samples. miR-187 was downregulated in colorectal cancer compared to normal controls, and its decreased expression tendency could also be detected in the plasma of CRC patients compared with healthy control patients. In a similar way, downregulation of miR-187 has been shown in renal cell carcinoma both in tissue and in plasma samples [56].

*In silico* target prediction of selected miRNAs, such as miR-31 and miR-4417 which are upregulated in tumor samples [57, 58], revealed a set of potential target mRNAs including Flavin containing monooxigenase 4 (FMO4), cyclin dependent kinase inhibitor 2B (CDKN2B), and prostaglandin D2 receptor (PTGDR), whose expression pattern changed oppositely  according to our (Human Transcriptome Array 2.0 microarray) results﻿. FMO4 is influenced by miR-31. FMO4 catalyzes NADPH-dependent oxidative metabolism and is downregulated in carcinoma cells by miR-31 [59]. Among the targets of miR-4417, PTGDR is downregulated in CRC which can be caused by promoter hypermethylation, as well [60].

“Previous studies reported that the highly conserved miR-195/497 cluster was significantly downregulated in gastric, breast, bladder, liver, and also in colorectal cancer [61–66]. Although, our investigation was limited to determine the expression level of miR-497 and its predicted target, CCND1 mRNA and protein level according to several functional analyses - such as luciferase assay-based methods - carried out in different types of cancers revealed that miR-497 (and miR-195) directly targets the 3’-UTR region of cyclin D1 [67–69]. Although, no experimental data is available to date about the interaction of miR-497-CCND1 in colorectal cancer, the overexpression of cyclin D1 mRNA might cause by the underexpression of miR-497 in colorectal adenoma and cancer by posttranscriptional silencing. Further investigations would confirmed our hypothesis.”

## Conclusion

We report here a study of the levels of 1733 mature miRNA in healthy, adenoma and colorectal cancer samples by two different methods. Besides the previously described miRNAs in pre- and neoplastic lesions, we also identified new, less known miRNAs (miR-4417), with altered expression in different colorectal neoplastic alterations. Our data also showed that tissue miRNA expression alterations could be observed also in plasma and may serve as valuable early diagnostic markers. Analysis of miRNA expression and predicted target mRNA was performed from the same patient set. Negative correlations were observed between miRNAs and targeted mRNAs, which could be explored further in functional analysis of neoplastic development related alterations.

## Additional files

Additional file 1: Clinicopathological features of 60 patients. (DOCX 18 kb)
Additional file 2: Detailed LogFC values between patient groups. (DOCX 24 kb)
Additional file 3: KEGG and GO analyses of the selected miRNAs. (DOCX 27 kb)

## ᅟ

ABHD2: Abhydrolase domain containing 2
ACAA2: Acetyl-CoA acyltransferase 2
ADtub/ADT: Tubular adenoma
ADtubvill/ADTV: Tubulovillous adenoma
ANXA1: Annexin 1
AXIN2: Axin 2
CALU: Calumenin
CCND1: Cyclin D1
CDK4: Cyclin dependent kinase 4
CDKN2B: Cyclin dependent kinase inhibitor 2B
CDKN2b: Cyclin-dependent kinase inhibitor 2B
CEP55: Centrosomal protein 55
CES2: Carboxylesterase 2
CRC/C: Colorectal cancer
DNAJC10: DnaJ heat shock protein family (Hsp40) member C10
E2F3: E2F transcription factor 3
FF: Fresh frozen
FFPET: Formalin-fixed, paraffin-embedded tissue
FMO4: Flavin containing monooxygenase 4
FSCN1: Fascin actin-bundling protein 1
GPRC5A: G protein-coupled receptor class C group 5 member A
ITGA2: Integrin subunit alpha 2
KIF5B: Kinesin family member 5B
L1CAM: L1 adhesion molecule
LIM: Lin11/Isl1/Mec3
miRNA: microRNA
N: Normal
NR5A2: Nuclear receptor subfamily 5 group A member 2
OSBPL3: Oxysterol binding protein like 3
PDE3A: Phosphodiesterase 3A
PPIL1: Peptidylprolyl isomerase like 1
PSAT1: Phosphoserine aminotransferase 1
PTGDR: Prostaglandin D2 receptor
RMA: Robust multichip averaging
RTN4: Reticulon 4
SESTD1: SEC14 and spectrin domain containing 1
SH3: SRC homology 3 d﻿omain
SNRPB2: Small nuclear ribonucleoprotein polypeptide B2
ST6GALNAC6: ST6 N-acetylgalactosaminide alpha-2,6-sialyltransferase 6
TESK2: Testis-specific kinase 2
TRANK1: Tetratricopeptide repeat and ankyrin repeat containing 1
UBE2H: Ubiquitin conjugating enzyme E2 H
ZNF391: Zinc finger protein 391
