# Supplementary material for: Colorectal adenoma and carcinoma specific miRNA profiles in biopsy and their expression in plasma specimens
Source: Clin Epigenetics. 2017 Feb 14;9:22. doi: 10.1186/s13148-016-0305-3 (PMC5310023; doi:10.1186/s13148-016-0305-3)
Supplement: Additional file 1: — Clinicopathological features of 60 patients. (DOCX 18 kb) [file 13148_2016_305_MOESM1_ESM.docx]

Additional file 1

Table S1. Clinicopathological features of 60 patients.

|  | **control (n=20)** | **adenoma (n=20)** | **colon cancer (n=20)** |
| --- | --- | --- | --- |
|  |  |  |  |
|  |  |  |  |
| **age, years, mean +- SD** | 51±17 | 64±10 | 63±14 |
|  |  |  |  |
| **gender, n (%)** |  |  |  |
| **female** | 16 (80%) | 10 (50%) | 8 (40%) |
| **male** | 4 (20%) | 10 (50%) | 12 (60%) |
| **colon cancer location n (%)** |  |  |  |
| **sigmoid** |  |  | 5 (25%) |
| **descending** |  |  | 1 (5%) |
| **transverse** |  |  | 2 (10%) |
| **ascending** |  |  | 1 (5%) |
| **cecum** |  |  | 1(5%) |
| **rectum** |  |  | 9 (45%) |
| **N/A** |  |  | 1(5%) |
| **colon cancer TNM stage, n (%)** |  |  |  |
| **I** |  |  | 1 (5%) |
| **II** |  |  | 7 (35%) |
| **III** |  |  | 9 (45%) |
| **IV** |  |  | 2 (10%) |
| **N/A** |  |  | 1 (5%) |
| **colon cancer DUKES stage, n (%)** |  |  |  |
| **DUKES B** |  |  | 6 (30%) |
| **DUKES C** |  |  | 9 (45%) |
| **DUKES D** |  |  | 3 (15%) |
| **N/A** |  |  | 2 (10%) |
| **adenoma features, n (%)** |  |  |  |
| **tubular** |  | 11 (55%) |  |
| **tubulovillous** |  | 9 (45%) |  |
| **adenoma location , n (%)**  **(per patient)** |  |  |  |
| **rectum** |  | 6 (30%) |  |
| **sigmoid** |  | 6 (30%) |  |
| **transverse** |  | 1 (5%) |  |
| **ascending** |  | 3 (15%) |  |
| **cecum** |  | 4 (20%) |  |
