# Supplementary material for: Colorectal adenoma and carcinoma specific miRNA profiles in biopsy and their expression in plasma specimens
Source: Clin Epigenetics. 2017 Feb 14;9:22. doi: 10.1186/s13148-016-0305-3 (PMC5310023; doi:10.1186/s13148-016-0305-3)
Supplement: Additional file 2: — Detailed LogFC values between patient groups. (DOCX 24 kb) [file 13148_2016_305_MOESM2_ESM.docx]

Additional file 2

Table S2. Detailed LogFC values between patient groups.

| **mIRNA ID** | **Normal versus CRC**  **P value Log_2_ FC** | |
| --- | --- | --- |
| **hsa-miR-31_st** | 8,6E-07 | -3,02 |
| **hsa-miR-4417_st** | 1,6E-07 | -2,45 |
| **hsa-miR-503_st** | 3,2E-09 | -2,23 |
| **hsa-miR-3647-5p_st** | 2,4E-04 | -1,67 |
| **hsa-miR-18a_st** | 2,3E-07 | -1,64 |
| **hsa-miR-4730_st** | 2,2E-04 | -1,58 |
| **hsa-miR-223_st** | 4,8E-04 | -1,42 |
| **hsa-miR-424-star_st** | 3,6E-07 | -1,40 |
| **hsa-miR-27a-star_st** | 4,2E-07 | -1,39 |
| **hsa-miR-29b-1-star_st** | 2,2E-05 | -1,39 |
| **hsa-miR-431_st** | 3,1E-07 | -1,39 |
| **hsa-miR-183-star_st** | 1,4E-05 | -1,36 |
| **hsa-miR-224_st** | 9,8E-07 | -1,34 |
| **hsa-miR-3687_st** | 5,2E-04 | -1,33 |
| **hsa-miR-1246_st** | 2,8E-04 | -1,30 |
| **hsa-miR-21-star_st** | 1,1E-06 | -1,26 |
| **hsa-miR-182_st** | 4,3E-05 | -1,23 |
| **hsa-miR-106b-star_st** | 5,7E-07 | -1,19 |
| **hsa-miR-4734_st** | 7,3E-04 | -1,19 |
| **hsa-miR-3613-3p_st** | 2,8E-03 | -1,18 |
| **hsa-miR-18b_st** | 1,0E-05 | -1,17 |
| **hsa-miR-3663-3p_st** | 1,2E-03 | -1,17 |
| **hsa-miR-4446-5p_st** | 2,2E-02 | -1,14 |
| **hsa-miR-3185_st** | 2,0E-03 | -1,12 |
| **hsa-miR-34b_st** | 2,1E-03 | -1,12 |
| **hsa-miR-584_st** | 6,1E-05 | -1,11 |
| **hsa-miR-31-star_st** | 1,1E-03 | -1,10 |
| **hsa-miR-20b_st** | 4,5E-06 | -1,09 |
| **hsa-miR-20a_st** | 5,0E-07 | -1,08 |
| **hsa-miR-4674_st** | 8,2E-04 | -1,08 |
| **hsa-miR-4741_st** | 7,4E-04 | -1,08 |
| **hsa-miR-106a_st** | 9,8E-07 | -1,06 |
| **hsa-miR-17_st** | 1,1E-06 | -1,05 |
| **hsa-miR-4668-5p_st** | 1,7E-03 | -1,05 |
| **hsa-miR-4640-5p_st** | 1,5E-04 | -1,04 |
| **hsa-miR-23a-star_st** | 4,7E-06 | -1,04 |
| **hsa-miR-708_st** | 5,8E-03 | -1,03 |
| **hsa-miR-3178_st** | 6,0E-04 | -1,01 |
| **hsa-miR-493_st** | 5,8E-06 | -1,01 |
| **hsa-miR-3591-3p_st** | 5,6E-03 | -1,01 |
| **hsa-miR-145_st** | 1,5E-04 | 1,01 |
| **hsa-miR-138_st** | 3,4E-03 | 1,02 |
| **hsa-miR-10a-star_st** | 2,9E-03 | 1,02 |
| **hsa-miR-154_st** | 9,2E-04 | 1,05 |
| **hsa-miR-149_st** | 3,8E-04 | 1,07 |
| **hsa-miR-299-5p_st** | 4,4E-03 | 1,08 |
| **hsa-miR-10a_st** | 4,9E-05 | 1,09 |
| **hsa-miR-30e-star_st** | 1,5E-07 | 1,10 |
| **hsa-miR-4645-5p_st** | 4,9E-06 | 1,10 |
| **hsa-miR-376c_st** | 8,2E-03 | 1,13 |
| **hsa-miR-664-star_st** | 1,7E-06 | 1,19 |
| **hsa-miR-29c-star_st** | 1,7E-04 | 1,26 |
| **hsa-miR-192-star_st** | 1,3E-04 | 1,27 |
| **hsa-miR-1_st** | 1,1E-04 | 1,28 |
| **hsa-miR-147b_st** | 5,4E-07 | 1,30 |
| **hsa-miR-342-5p_st** | 1,8E-05 | 1,31 |
| **hsa-miR-497_st** | 6,1E-06 | 1,33 |
| **hsa-miR-378_st** | 3,3E-07 | 1,35 |
| **hsa-miR-195_st** | 1,3E-06 | 1,38 |
| **hsa-miR-194-star_st** | 3,2E-05 | 1,44 |
| **hsa-miR-378c_st** | 2,9E-07 | 1,47 |
| **hsa-miR-378g_st** | 2,6E-08 | 1,52 |
| **hsa-miR-378-star_st** | 3,6E-08 | 1,53 |
| **hsa-miR-30a-star_st** | 3,9E-06 | 1,54 |
| **hsa-miR-378f_st** | 1,3E-07 | 1,55 |
| **hsa-miR-378d_st** | 2,6E-08 | 1,56 |
| **hsa-miR-196b_st** | 1,5E-04 | 1,57 |
| **hsa-miR-378i_st** | 1,2E-07 | 1,58 |
| **hsa-miR-378e_st** | 8,4E-07 | 1,58 |
| **hsa-miR-10b-star_st** | 2,8E-09 | 1,65 |
| **hsa-miR-422a_st** | 1,6E-07 | 1,66 |
| **hsa-miR-150_st** | 7,6E-09 | 1,67 |
| **hsa-miR-196a_st** | 8,0E-04 | 1,70 |
| **hsa-miR-133b_st** | 5,8E-08 | 1,80 |
| **hsa-miR-10b_st** | 1,2E-08 | 1,90 |
| **hsa-miR-139-5p_st** | 1,0E-09 | 1,95 |
| **hsa-miR-215_st** | 1,6E-05 | 2,08 |
| **hsa-miR-133a_st** | 9,8E-08 | 2,17 |
| **hsa-miR-375_st** | 4,2E-05 | 2,21 |

Abbrevations: CRC, colorectal cancer

| **miRNA ID** | **ADtub versus ADtubvill P value Log_2_ FC** | |
| --- | --- | --- |
| **hsa-miR-489_st** | 3,0E-03 | -2,26 |
| **hsa-miR-183_st** | 1,0E-02 | -1,58 |
| **hsa-miR-374b_st** | 9,3E-03 | -1,22 |
| **hsa-miR-335_st** | 2,0E-03 | -1,18 |
| **hsa-miR-338-5p_st** | 5,1E-03 | -1,16 |
| **hsa-miR-96_st** | 1,2E-02 | -1,15 |
| **hsa-miR-552_st** | 1,4E-02 | -1,13 |
| **hsa-miR-138-1-star_st** | 3,2E-02 | -1,08 |
| **hsa-miR-183-star_st** | 1,5E-02 | -1,08 |
| **hsa-miR-3175_st** | 5,6E-03 | 1,38 |
| **hsa-miR-720_st** | 2,6E-02 | 1,37 |
| **hsa-miR-4492_st** | 1,9E-02 | 1,10 |
| **hsa-miR-4508_st** | 2,1E-02 | 1,02 |

Abbrevations: AD_tub_, tubular adenoma; AD_tubvill_, tubulovillous adenoma

| **miRNA ID** | **N versus Ad**  **P value Log2 FC** | |
| --- | --- | --- |
| **hsa-miR-31_st** | 3,1E-05 | -3,05 |
| **hsa-miR-183-star_st** | 1,3E-09 | -2,10 |
| **hsa-miR-182_st** | 7,6E-11 | -1,93 |
| **hsa-miR-31-star_st** | 2,8E-03 | -1,57 |
| **hsa-miR-183_st** | 7,6E-05 | -1,57 |
| **hsa-miR-584_st** | 8,6E-09 | -1,51 |
| **hsa-miR-4417_st** | 5,7E-04 | -1,50 |
| **hsa-miR-96_st** | 1,9E-06 | -1,45 |
| **hsa-miR-34a-star_st** | 3,0E-06 | -1,34 |
| **hsa-miR-29b-1-star_st** | 1,3E-04 | -1,31 |
| **hsa-miR-27a-star_st** | 7,5E-08 | -1,31 |
| **hsa-miR-34a_st** | 3,2E-09 | -1,25 |
| **hsa-miR-21-star_st** | 4,1E-09 | -1,17 |
| **hsa-miR-592_st** | 4,5E-03 | -1,15 |
| **hsa-miR-4449_st** | 9,6E-05 | -1,13 |
| **hsa-miR-625_st** | 1,2E-07 | -1,10 |
| **hsa-miR-3663-3p_st** | 5,8E-04 | -1,05 |
| **hsa-miR-224_st** | 1,7E-05 | -1,04 |
| **hsa-miR-720_st** | 1,1E-02 | -1,02 |
| **hsa-miR-181c-star_st** | 1,1E-05 | -1,01 |
| **hsa-miR-135b-star_st** | 5,6E-08 | -1,00 |
| **hsa-miR-299-5p_st** | 1,1E-05 | 1,62 |
| **hsa-miR-376c_st** | 6,4E-06 | 1,53 |
| **hsa-miR-215_st** | 1,1E-07 | 1,52 |
| **hsa-miR-10b_st** | 2,7E-08 | 1,48 |
| **hsa-miR-133b_st** | 4,7E-06 | 1,42 |
| **hsa-miR-133a_st** | 4,3E-05 | 1,38 |
| **hsa-miR-1_st** | 5,1E-05 | 1,36 |
| **hsa-miR-139-5p_st** | 3,7E-09 | 1,31 |
| **hsa-miR-195_st** | 4,4E-07 | 1,29 |
| **hsa-miR-150_st** | 1,6E-06 | 1,23 |
| **hsa-miR-10b-star_st** | 8,0E-07 | 1,21 |
| **hsa-miR-381_st** | 1,1E-06 | 1,20 |
| **hsa-miR-99a_st** | 5,8E-05 | 1,07 |
| **hsa-miR-154_st** | 8,1E-06 | 1,06 |
| **hsa-miR-9-star_st** | 9,1E-06 | 1,02 |
| **hsa-miR-199b-3p_st** | 6,7E-08 | 1,01 |
| **hsa-miR-199a-3p_st** | 6,6E-08 | 1,00 |

Abbrevations: N, normal; Ad, Adenoma
