# Supplementary material for: Colorectal adenoma and carcinoma specific miRNA profiles in biopsy and their expression in plasma specimens
Source: Clin Epigenetics. 2017 Feb 14;9:22. doi: 10.1186/s13148-016-0305-3 (PMC5310023; doi:10.1186/s13148-016-0305-3)
Supplement: Additional file 3: — KEGG and GO analyses of the selected miRNAs. (DOCX 27 kb) [file 13148_2016_305_MOESM3_ESM.docx]

Additional file 3

Table S3. KEGG pathways of the continuously changing miRNAs in expression

| [**KEGG PATHWAY**](https://david.ncifcrf.gov/chartReport.jsp?d-16544-s=2&d-16544-o=2&annot=55&d-16544-p=1) | **Gene number percentage (%)** | [**P-Value**](https://david.ncifcrf.gov/chartReport.jsp?d-16544-s=7&d-16544-o=1&annot=55&d-16544-p=1) |
| --- | --- | --- |
| [**Pathways in cancer**](https://david.ncifcrf.gov/kegg.jsp?path=hsa05200$Pathways%20in%20cancer&termId=550028893&source=kegg) | 10,7 | 6,4E-07 |
| [**PI3K-Akt signaling pathway**](https://david.ncifcrf.gov/kegg.jsp?path=hsa04151$PI3K-Akt%20signaling%20pathway&termId=550028768&source=kegg) | 9,0 | 1,70E-05 |
| [**MAPK signaling pathway**](https://david.ncifcrf.gov/kegg.jsp?path=hsa04010$MAPK%20signaling%20pathway&termId=550028740&source=kegg) | 7,9 | 6,4E-07 |
| [**Rap1 signaling pathway**](https://david.ncifcrf.gov/kegg.jsp?path=hsa04015$Rap1%20signaling%20pathway&termId=550028743&source=kegg) | 7,4 | 3,2E-08 |
| [**Proteoglycans in cancer**](https://david.ncifcrf.gov/kegg.jsp?path=hsa05205$Proteoglycans%20in%20cancer&termId=550028897&source=kegg) | 7,0 | 6,2E-08 |
| [**cAMP signaling pathway**](https://david.ncifcrf.gov/kegg.jsp?path=hsa04024$cAMP%20signaling%20pathway&termId=550028746&source=kegg) | 6,7 | 3,9E-07 |
| [**Focal adhesion**](https://david.ncifcrf.gov/kegg.jsp?path=hsa04510$Focal%20adhesion&termId=550028783&source=kegg) | 6,6 | 3,00E-06 |
| [**MicroRNAs in cancer**](https://david.ncifcrf.gov/kegg.jsp?path=hsa05206$MicroRNAs%20in%20cancer&termId=550028898&source=kegg) | 6,6 | 3,50E-03 |
| [**Ras signaling pathway**](https://david.ncifcrf.gov/kegg.jsp?path=hsa04014$Ras%20signaling%20pathway&termId=550028742&source=kegg) | 6,4 | 6,60E-05 |
| [**HTLV-I infection**](https://david.ncifcrf.gov/kegg.jsp?path=hsa05166$HTLV-I%20infection&termId=550028890&source=kegg) | 6,2 | 1,70E-03 |
| [**Endocytosis**](https://david.ncifcrf.gov/kegg.jsp?path=hsa04144$Endocytosis&termId=550028764&source=kegg) | 6,1 | 3,50E-03 |
| [**cGMP-PKG signaling pathway**](https://david.ncifcrf.gov/kegg.jsp?path=hsa04022$cGMP-PKG%20signaling%20pathway&termId=550028745&source=kegg) | 5,9 | 7,3E-07 |
| [**Regulation of actin cytoskeleton**](https://david.ncifcrf.gov/kegg.jsp?path=hsa04810$Regulation%20of%20actin%20cytoskeleton&termId=550028824&source=kegg) | 5,9 | 1,70E-04 |
| [**Oxytocin signaling pathway**](https://david.ncifcrf.gov/kegg.jsp?path=hsa04921$Oxytocin%20signaling%20pathway&termId=550028836&source=kegg) | 5,6 | 1,9E-06 |
| [**Axon guidance**](https://david.ncifcrf.gov/kegg.jsp?path=hsa04360$Axon%20guidance&termId=550028779&source=kegg) | 5,1 | 3,6E-07 |
| [**Signaling pathways regulating pluripotency of stem cells**](https://david.ncifcrf.gov/kegg.jsp?path=hsa04550$Signaling%20pathways%20regulating%20pluripotency%20of%20stem%20cells&termId=550028789&source=kegg) | 4,8 | 2,70E-05 |
| [**Adrenergic signaling in cardiomyocytes**](https://david.ncifcrf.gov/kegg.jsp?path=hsa04261$Adrenergic%20signaling%20in%20cardiomyocytes&termId=550028772&source=kegg) | 4,8 | 5,90E-05 |
| [**Hippo signaling pathway**](https://david.ncifcrf.gov/kegg.jsp?path=hsa04390$Hippo%20signaling%20pathway&termId=550028782&source=kegg) | 4,8 | 1,10E-04 |
| [**Neurotrophin signaling pathway**](https://david.ncifcrf.gov/kegg.jsp?path=hsa04722$Neurotrophin%20signaling%20pathway&termId=550028812&source=kegg) | 4,6 | 3,8E-06 |
| [**Oocyte meiosis**](https://david.ncifcrf.gov/kegg.jsp?path=hsa04114$Oocyte%20meiosis&termId=550028756&source=kegg) | 4,4 | 1,8E-06 |
| [**Viral carcinogenesis**](https://david.ncifcrf.gov/kegg.jsp?path=hsa05203$Viral%20carcinogenesis&termId=550028895&source=kegg) | 4,3 | 5,90E-02 |
| [**Dopaminergic synapse**](https://david.ncifcrf.gov/kegg.jsp?path=hsa04728$Dopaminergic%20synapse&termId=550028818&source=kegg) | 4,1 | 2,80E-04 |
| [**Insulin signaling pathway**](https://david.ncifcrf.gov/kegg.jsp?path=hsa04910$Insulin%20signaling%20pathway&termId=550028825&source=kegg) | 4,1 | 8,80E-04 |
| [**Calcium signaling pathway**](https://david.ncifcrf.gov/kegg.jsp?path=hsa04020$Calcium%20signaling%20pathway&termId=550028744&source=kegg) | 4,1 | 2,40E-02 |

Table S4. GO BP analysis of the continuously changing miRNAs inexpression

| [**GO BP analysis**](https://david.ncifcrf.gov/chartReport.jsp?d-16544-s=2&d-16544-o=2&annot=55&d-16544-p=1) | **Gene number percentage (%)** | [**P-Value**](https://david.ncifcrf.gov/chartReport.jsp?d-16544-s=7&d-16544-o=1&annot=30&d-16544-p=1) |
| --- | --- | --- |
| [**transcription, DNA-templated**](http://www.ebi.ac.uk/QuickGO/GTerm?id=GO:0006351) | 16,9 | 4,40E-10 |
| [**positive regulation of transcription from RNA polymerase II promoter**](http://www.ebi.ac.uk/QuickGO/GTerm?id=GO:0045944) | 12,8 | 1,30E-24 |
| [**regulation of transcription, DNA-templated**](http://www.ebi.ac.uk/QuickGO/GTerm?id=GO:0006355) | 12,3 | 1,30E-05 |
| [**negative regulation of transcription from RNA polymerase II promoter**](http://www.ebi.ac.uk/QuickGO/GTerm?id=GO:0000122) | 10,2 | 8,70E-23 |
| [**signal transduction**](http://www.ebi.ac.uk/QuickGO/GTerm?id=GO:0007165) | 9,4 | 1,70E-04 |
| [**transcription from RNA polymerase II promoter**](http://www.ebi.ac.uk/QuickGO/GTerm?id=GO:0006366) | 7,2 | 5,50E-16 |
| [**positive regulation of transcription, DNA-templated**](http://www.ebi.ac.uk/QuickGO/GTerm?id=GO:0045893) | 7,1 | 1,20E-15 |
| [**protein phosphorylation**](http://www.ebi.ac.uk/QuickGO/GTerm?id=GO:0006468) | 5,7 | 1,70E-10 |
| [**negative regulation of transcription, DNA-templated**](http://www.ebi.ac.uk/QuickGO/GTerm?id=GO:0045892) | 5,7 | 1,20E-08 |
| [**apoptotic process**](http://www.ebi.ac.uk/QuickGO/GTerm?id=GO:0006915) | 4,6 | 9,20E-03 |
| [**negative regulation of cell proliferation**](http://www.ebi.ac.uk/QuickGO/GTerm?id=GO:0008285) | 4,5 | 3,10E-06 |
| [**regulation of transcription from RNA polymerase II promoter**](http://www.ebi.ac.uk/QuickGO/GTerm?id=GO:0006357) | 4,5 | 2,60E-05 |
| [**positive regulation of cell proliferation**](http://www.ebi.ac.uk/QuickGO/GTerm?id=GO:0008284) | 4,5 | 4,20E-04 |
| [**cell adhesion**](http://www.ebi.ac.uk/QuickGO/GTerm?id=GO:0007155) | 4,3 | 3,70E-04 |
| [**intracellular signal transduction**](http://www.ebi.ac.uk/QuickGO/GTerm?id=GO:0035556) | 4,1 | 8,00E-05 |
| [**negative regulation of apoptotic process**](http://www.ebi.ac.uk/QuickGO/GTerm?id=GO:0043066) | 4,0 | 5,70E-03 |
| [**cell proliferation**](http://www.ebi.ac.uk/QuickGO/GTerm?id=GO:0008283) | 3,7 | 2,00E-04 |
| [**positive regulation of apoptotic process**](http://www.ebi.ac.uk/QuickGO/GTerm?id=GO:0043065) | 3,3 | 1,00E-04 |
| [**protein ubiquitination**](http://www.ebi.ac.uk/QuickGO/GTerm?id=GO:0016567) | 3,3 | 2,20E-03 |
| [**nervous system development**](http://www.ebi.ac.uk/QuickGO/GTerm?id=GO:0007399) | 3,1 | 1,10E-04 |
| [**axon guidance**](http://www.ebi.ac.uk/QuickGO/GTerm?id=GO:0007411) | 2,9 | 4,50E-10 |
| [**chemical synaptic transmission**](http://www.ebi.ac.uk/QuickGO/GTerm?id=GO:0007268) | 2,9 | 3,80E-05 |
| [**positive regulation of gene expression**](http://www.ebi.ac.uk/QuickGO/GTerm?id=GO:0010628) | 2,7 | 6,10E-04 |

Table S5. KEGG pathways of the 23 miRNAs altered between normal vs. adenoma and CRC samples

| [**KEGG PATHWAY**](https://david.ncifcrf.gov/chartReport.jsp?d-16544-s=2&d-16544-o=2&annot=55&d-16544-p=1) | **Gene number percentage (%)** | [**P-Value**](https://david.ncifcrf.gov/chartReport.jsp?d-16544-s=7&d-16544-o=1&annot=55&d-16544-p=1) |
| --- | --- | --- |
| **Pathways in cancer** | 11,9 | 3,41E-17 |
| **PI3K-Akt signaling pathway** | 9,2 | 8,18E-10 |
| **MAPK signaling pathway** | 7,8 | 7,21E-12 |
| **Endocytosis** | 7,0 | 5,64E-08 |
| **HTLV-I infection** | 6,9 | 9,18E-08 |
| **Proteoglycans in cancer** | 6,7 | 3,96E-12 |
| **Focal adhesion** | 6,3 | 1,27E-09 |
| **Ras signaling pathway** | 6,2 | 1,76E-07 |
| **MicroRNAs in cancer** | 6,0 | 1,46E-03 |
| **Rap1 signaling pathway** | 5,9 | 1,28E-07 |
| **Regulation of actin cytoskeleton** | 5,7 | 8,56E-07 |
| **Axon guidance** | 5,6 | 1,03E-16 |
| **cAMP signaling pathway** | 5,3 | 5,69E-06 |
| **Wnt signaling pathway** | 5,0 | 1,65E-10 |
| **Signaling pathways regulating pluripotency of stem cells** | 5,0 | 3,02E-10 |
| **cGMP-PKG signaling pathway** | 5,0 | 2,07E-07 |
| **Hippo signaling pathway** | 4,9 | 1,86E-08 |
| **Oxytocin signaling pathway** | 4,9 | 9,67E-08 |
| **Adrenergic signaling in cardiomyocytes** | 4,7 | 4,64E-08 |
| **Neurotrophin signaling pathway** | 4,6 | 8,65E-11 |
| **Transcriptional misregulation in cancer** | 4,5 | 2,50E-05 |
| **Calcium signaling pathway** | 4,4 | 2,73E-04 |
| **Chemokine signaling pathway** | 4,4 | 6,72E-04 |
| **FoxO signaling pathway** | 4,2 | 5,00E-07 |
| **Hepatitis B** | 4,2 | 5,22E-06 |
| **Oocyte meiosis** | 4,1 | 1,59E-09 |
| **Dopaminergic synapse** | 4,1 | 3,43E-07 |

Table S6. Go BP analysis of the 23 miRNAs altered between normal vs. adenoma and CRC samples

| [**GO BP analysis**](https://david.ncifcrf.gov/chartReport.jsp?d-16544-s=2&d-16544-o=2&annot=55&d-16544-p=1) | **Gene number percentage (%)** | [**P-Value**](https://david.ncifcrf.gov/chartReport.jsp?d-16544-s=7&d-16544-o=1&annot=30&d-16544-p=1) |
| --- | --- | --- |
| **transcription, DNA-templated** | 16,5 | 1,78E-14 |
| **positive regulation of transcription from RNA polymerase II promoter** | 12,2 | 3,84E-38 |
| **regulation of transcription, DNA-templated** | 12,2 | 7,64E-09 |
| **signal transduction** | 9,0 | 1,74E-05 |
| **negative regulation of transcription from RNA polymerase II promoter** | 8,5 | 1,25E-22 |
| **positive regulation of transcription, DNA-templated** | 7,1 | 3,71E-28 |
| **transcription from RNA polymerase II promoter** | 6,4 | 7,19E-20 |
| **positive regulation of GTPase activity** | 5,5 | 3,97E-09 |
| **protein phosphorylation** | 5,2 | 1,78E-13 |
| **negative regulation of transcription, DNA-templated** | 4,9 | 1,45E-08 |
| **positive regulation of cell proliferation** | 4,5 | 1,64E-06 |
| **apoptotic process** | 4,4 | 2,42E-03 |
| **negative regulation of cell proliferation** | 4,3 | 7,78E-09 |
| **intracellular signal transduction** | 4,2 | 1,39E-08 |
| **negative regulation of apoptotic process** | 4,2 | 1,74E-05 |
| **cell adhesion** | 4,1 | 1,71E-05 |
| **regulation of transcription from RNA polymerase II promoter** | 3,9 | 2,85E-05 |
| **nervous system development** | 3,8 | 5,09E-14 |
| **multicellular organism development** | 3,8 | 4,23E-02 |
| **protein transport** | 3,1 | 1,09E-02 |
| **cell proliferation** | 3,0 | 4,90E-03 |
| **MAPK cascade** | 2,8 | 9,44E-07 |
| **positive regulation of apoptotic process** | 2,8 | 4,35E-04 |
| **viral process** | 2,8 | 2,72E-03 |
| **cell division** | 2,8 | 8,93E-03 |
| **protein ubiquitination** | 2,8 | 1,95E-02 |
| **axon guidance** | 2,7 | 4,66E-16 |

Table S7. KEGG pathways of the 4 selected adenoma specific miRNAs

| [**KEGG PATHWAY**](https://david.ncifcrf.gov/chartReport.jsp?d-16544-s=2&d-16544-o=2&annot=55&d-16544-p=1) | **Gene number percentage (%)** | [**P-Value**](https://david.ncifcrf.gov/chartReport.jsp?d-16544-s=7&d-16544-o=1&annot=55&d-16544-p=1) |
| --- | --- | --- |
| **Pathways in cancer** | 12,3 | 4,87E-07 |
| **Rap1 signaling pathway** | 7,7 | 5,47E-06 |
| **MAPK signaling pathway** | 7,7 | 2,11E-04 |
| **MicroRNAs in cancer** | 7,2 | 5,20E-03 |
| **Proteoglycans in cancer** | 6,9 | 4,91E-05 |
| **cAMP signaling pathway** | 6,4 | 2,85E-04 |
| **Ras signaling pathway** | 6,4 | 1,90E-03 |
| **Adrenergic signaling in cardiomyocytes** | 6,2 | 5,82E-06 |
| **cGMP-PKG signaling pathway** | 6,2 | 4,94E-05 |
| **HTLV-I infection** | 6,2 | 1,73E-02 |
| **Endocytosis** | 6,2 | 1,89E-02 |
| **Axon guidance** | 5,9 | 1,86E-06 |
| **Regulation of actin cytoskeleton** | 5,9 | 3,64E-03 |
| **Oxytocin signaling pathway** | 5,6 | 1,91E-04 |
| **Melanogenesis** | 5,4 | 5,20E-07 |
| **Glutamatergic synapse** | 5,1 | 1,68E-05 |
| **Focal adhesion** | 5,1 | 2,26E-02 |
| **Hippo signaling pathway** | 4,9 | 1,95E-03 |
| **Transcriptional misregulation in cancer** | 4,9 | 6,18E-03 |
| **Oocyte meiosis** | 4,6 | 1,10E-04 |
| **Dopaminergic synapse** | 4,6 | 7,76E-04 |
| **Signaling pathways regulating pluripotency of stem cells** | 4,6 | 2,11E-03 |
| **Chemokine signaling pathway** | 4,6 | 3,24E-02 |
| **Estrogen signaling pathway** | 4,4 | 1,14E-04 |
| **Calcium signaling pathway** | 4,4 | 4,42E-02 |
| **Aldosterone synthesis and secretion** | 4,1 | 3,67E-05 |
| **Circadian entrainment** | 4,1 | 2,41E-04 |
| **Cholinergic synapse** | 4,1 | 1,30E-03 |
| **Sphingolipid signaling pathway** | 4,1 | 2,86E-03 |
| **Platelet activation** | 4,1 | 6,13E-03 |

Table S8. GO Bp analysis of the 4 selected adenoma specific miRNAs

| **GO BP analysis** | **Gene number percentage %** | **P-value** |
| --- | --- | --- |
| **transcription, DNA-templated** | 14,7 | 3,32E-03 |
| **positive regulation of transcription from RNA polymerase II promoter** | 13,2 | 5,31E-17 |
| **regulation of transcription, DNA-templated** | 10,7 | 5,81E-02 |
| **signal transduction** | 9,0 | 1,36E-02 |
| **negative regulation of transcription from RNA polymerase II promoter** | 8,6 | 1,38E-08 |
| **positive regulation of transcription, DNA-templated** | 7,8 | 1,09E-12 |
| **transcription from RNA polymerase II promoter** | 6,7 | 2,31E-08 |
| **positive regulation of GTPase activity** | 5,9 | 7,65E-05 |
| **negative regulation of transcription, DNA-templated** | 5,7 | 1,22E-05 |
| **intracellular signal transduction** | 4,8 | 3,18E-05 |
| **positive regulation of cell proliferation** | 4,8 | 1,11E-03 |
| **nervous system development** | 4,5 | 7,11E-08 |
| **regulation of transcription from RNA polymerase II promoter** | 4,5 | 1,08E-03 |
| **apoptotic process** | 4,5 | 6,24E-02 |
| **negative regulation of cell proliferation** | 4,4 | 5,88E-04 |
| **protein transport** | 4,3 | 6,87E-04 |
| **protein phosphorylation** | 4,3 | 7,24E-03 |
| **multicellular organism development** | 4,1 | 7,36E-02 |
| **negative regulation of apoptotic process** | 3,8 | 5,83E-02 |
| **positive regulation of cell migration** | 3,3 | 7,17E-07 |
| **chemical synaptic transmission** | 3,1 | 2,20E-04 |
| **small GTPase mediated signal transduction** | 3,0 | 7,42E-04 |
| **transport** | 3,0 | 5,51E-02 |
